# P-1072. Evaluation of the Spontaneous Mutation Frequencies of SPR719 Alone and in Combination with Other Agents Used to Treat *Mycobacterium avium* Complex Pulmonary Disease

**DOI:** 10.1093/ofid/ofae631.1260

**Published:** 2025-01-29

**Authors:** Chris Pillar, Olivia Walser, Naomi DeLaet, Zach Leer, Mary Thwaites, David A Hufnagel, Nicole Cotroneo, Ian A Critchley, Kamal Hamed

**Affiliations:** Micromyx, Kalamazoo, Michigan; Microbiologics, Kalamazoo, Michigan; Microbiologics, Kalamazoo, Michigan; Microbiologics, Kalamazoo, Michigan; microbiologics, kalamazoo, Michigan; Microbiologics, Kalamazoo, Michigan; Spero Therapeutics, Cambridge, Massachusetts; Spero Therapeutics, Cambridge, Massachusetts; Spero Therapeutics, Inc., Cambridge, Massachusetts

## Abstract

**Background:**

SPR720 (fobrepodacin), a novel oral benzimidazole prodrug that inhibits the ATPase activity of mycobacterial DNA gyrase B, is being developed for treatment of nontuberculous mycobacterial pulmonary disease (NTM-PD). SPR719, the active moiety of SPR720, has previously demonstrated antimycobacterial activity against nontuberculous mycobacteria (NTM), including *Mycobacterium avium* complex (MAC), the most common causative agent of NTM-PD.

**Methods:**

**In this study, the spontaneous mutation frequencies (SMFs) of MAC ATCC 700898 (macrolide-susceptible) and a clinical macrolide-resistant MAC isolate (MMX 9461) were evaluated in the presence of SPR719 alone, standard-of-care antimycobacterial agents (clarithromycin, ethambutol, and rifampin) alone, and for SPR719 in combination with these agents.** The minimal inhibitory concentrations (MICs) for SPR719 and comparators against the NTM were determined by agar dilution. SMFs were determined by plating a high inoculum (10^8^-10^9^ colony forming units) on selective agar prepared at 2-, 4-, and 8-fold the MIC for SPR719, and at 4-fold the MIC for comparator agents. SMFs were also determined for SPR719 in combination with antimycobacterial agents where SPR719 was evaluated at 2-fold the MIC and a paired agent was evaluated at 4-fold the MIC. Putative mutants were patched from selection plates onto agar containing the selection drug, and susceptibility was then evaluated by broth microdilution alongside the parent isolate.

**Results:**

All concentrations of SPR719 alone and in combination with other agents prevented growth of both isolates, resulting in low SMFs for SPR719 (< 2.21 × 10^-9^ and < 7.14 × 10^-11^). In contrast, mutants were readily selected with rifampin (SMF of 2.55 × 10^-8^ and 1.99 × 10^-8^) and clarithromycin (SMF of 1.16 × 10^-8^), and these mutants exhibited elevated MIC values ≥ 4-fold the MIC of the parent by broth microdilution. Colonies emerged during selection with ethambutol but were unstable and did not grow when patched onto ethambutol-containing agar.

**Conclusion:**

The low propensity for the development of resistance against the novel agent SPR719 alone and in combination with other agents is important because emergence of resistance is a clinical concern in the setting of prolonged combination therapy for NTM-PD.

**Disclosures:**

**Nicole Cotroneo, B.S.**, Spero: Advisor/Consultant **Ian A. Critchley, PhD**, Spero: Employee **Kamal Hamed, MD, MPH**, Spero: Employee

